# Bimanual or unimanual stacking strategies under different cognitive loads: Evidence of a cognitive/action trade‐off in the coordination strategy of 3‐ to 5‐year‐olds

**DOI:** 10.1111/bjdp.70011

**Published:** 2025-08-28

**Authors:** Lisanne Schröer, Johanna‐Katharina Maninger, Richard P. Cooper, Denis Mareschal

**Affiliations:** ^1^ Centre for Brain and Cognitive Development Birkbeck, University of London London UK; ^2^ Centre for Cognition, Computation and Modelling Birkbeck, University of London London UK

**Keywords:** action planning, bimanual coordination, cognitive load, motor strategies, preschoolers

## Abstract

Previous studies have found that increased cognitive load during a task might result in the use of ‘easier’ motor strategies that nevertheless achieve task goals. Here, we investigated the influence of cognitive load on bimanual or unimanual strategy use in preschoolers, through a combination of secondary data analysis and new empirical data. Experiment 1 investigated block‐stacking strategies under high, medium and low cognitive load tasks in 3‐year‐olds and showed that 3‐year‐olds demonstrated significantly more unimanual strategy use in the high cognitive load task. Experiment 2 investigated (i) whether this effect persisted across preschool years and (ii) whether it was modulated by differences in executive function abilities. There was no age effect in motor strategy use under high cognitive load from 3 to 5 years of age. However, individual differences in inhibitory control and working memory use were significantly associated with differences in unimanual strategy use. These results are interpreted as evidence for a cognitive/action trade‐off in which higher cognitive demands result in the adjustment of motor strategies such as use of unimanual stacking instead of bimanual coordination in preschoolers.

**Statement of Contribution**

- This study examined the effect of cognitive load on motor coordination strategy during stacking actions in preschoolers.
- Three‐year‐olds showed more unimanual coordination strategy under high cognitive load compared to lower cognitive load, and this effect of high cognitive load on coordination strategy use remained persistent across preschool years.
- The use of coordination strategy under high cognitive load was modulated by differences in executive functions.
- The current results demonstrated a cognitive/action trade‐off in the adjustment of motor coordination strategy under increased cognitive load in preschoolers.

## INTRODUCTION

Countless everyday activities, such as cooking, typing or catching a ball, require bimanual coordination—the coordinated use of both hands to execute a movement or action (Fagard & Jacquet, 1989). Bimanual coordination is also the most efficient motor strategy in many tasks where a unimanual approach is possible, such as when stacking blocks on a flat surface. In general terms, bimanual coordination requires coordinated sensorimotor control between the two hands as an individual performs an action or sequence of actions (Babik & Michel, 2013). The first evidence of such coordination occurs around 6 to 10 months of age, involving, for example, banging two objects together (Ramsay, 1985). This symmetric form of bimanual coordination, where both hands have a similar role, normally precedes the emergence of asymmetrical bimanual coordination, where each hand completes a complementary role, as in using one hand to stabilize an object while using the other to stack a second object on top of the first (Fagard & Jacquet, 1989).

One type of asymmetrical bimanual coordination is role‐differentiated bimanual manipulation where each hand performs different, but complementary action(s) on one or more objects (Kimmerle et al., 2010). Infants exhibit initial evidence of role‐differentiated bimanual manipulation late in their first year of life (Bruner, 1970; Kimmerle et al., 2010; Ramsay & Weber, 1986). In about 80% of infants, simple role‐differentiated bimanual manipulation can be observed from about 7 months of age, although it does not occur often (Kimmerle et al., 1995). By 11 months of age, all typically developing infants show role‐differentiated bimanual manipulation at increasing frequency, such as when fingering and stroking an object (Fagard & Pezé, 1997; Kimmerle et al., 1995). By 18 months, infants are generally able to use both hands in different roles to retrieve objects (Birtles et al., 2011). More complex role‐differentiated bimanual manipulation such as unscrewing develops between 18 and 24 months of age (Fagard & Jacquet, 1989; Ramsay & Weber, 1986). Thus, by the end of the 2nd year of life, typically developing young children can coordinate their hands effectively and reliably to execute goal‐directed actions.

While younger children can coordinate their hands bimanually, adults are generally faster and more precise than children (Kuhtz‐Buschbeck et al., 1999; Marion et al., 2003; Mason et al., 2010, 2013; Ringenbach & Amazeen, 2005; Robertson, 2001; Steese‐Seda et al., 1995). Perfection and the development of more taxing forms of symmetrical and asymmetrical bimanual coordination occur later in childhood. Several external factors such as object size (Mason et al., 2010) and distance (Mason et al., 2013) have been found to influence bimanual control across childhood. However, cognitive factors, such as cognitive load, can also play a role in action control and execution (e.g., Engström et al., 2017). Thus, in the studies reported here, we aim to explore the interaction between action selection and cognitive load in early childhood. Specifically, we ask whether cognitive load plays a role in the choice between the motor strategies of bimanual coordination and unimanual control in early development, and consequently whether manual strategy (unimanual or bimanual) might be used as a marker of cognitive load.

Cognitive load can be defined as the ‘amount’ of cognitive resource demanded from an actor in an activity (e.g., Engström et al., 2017) and is operationalized in the current study in terms of the planning complexity of an otherwise identical (motor‐wise) stacking action. Cognitive load has been manipulated in various ways in previous studies: by using a dual‐task design (e.g., Bertrand & Camos, 2015; Boudreau & Bushnell, 2000; Sebastian & Hernández‐Gil, 2013), by manipulating motor difficulties necessary for the task (e.g., DeMasi & Berger, 2021; Smith et al., 2016), or as in the current study, by manipulating the context in which a motor task is executed (such as walking on flat ground vs. walking on a staircase; Berger, 2010).

Some evidence relevant to the interaction between cognitive factors and motor control across development has already been reported. For example, Boudreau and Bushnell (2000) showed that if a 10‐month‐old's mental resources were directed to thinking about a movement's goal‐state, then motor planning and execution were impaired (Boudreau & Bushnell, 2000). Similarly, 13‐month‐olds can inhibit a prepotent locomotor response when the cognitive task demands are low but perseverate if the task demands are high, in an A‐not‐B task (Berger, 2010). Additional trade‐offs between cognitive load and action efficacy in infancy have been reported across a range of similar tasks (e.g., Berger et al., 2018; Berthier et al., 2001; Hespos & Baillargeon, 2006; Keen et al., 2003).

Early childhood is a period that is characterized by dramatic improvements in executive functions and cognitive control (Anderson & Reidy, 2012; Diamond, 2013; Garon et al., 2008), but similar trade‐offs between cognitive load and motor control have also been reported over this period (Bertrand & Camos, 2015; DeMasi & Berger, 2021; Sebastian & Hernández‐Gil, 2013; Smith et al., 2016). For example, in a high motor‐demand task, Bertrand and Camos (2015) found impaired recall and execution of previously presented sequences in 4‐ to 6‐year‐olds, while cognitive load continues to impact motor behaviours such as postural sway (Blanchard et al., 2005; Igarashi et al., 2016; Olivier et al., 2008; Schmid et al., 2006) and tongue protrusions (Forrester & Rodriguez, 2015) throughout childhood.

However, even if the cognitive load of a task is high, some tasks afford the use of motor strategies that may lighten the motor load while still achieving the goal, and infants and children can adopt these strategies when cognitive load is high. For example, infants may use one arm to aid sitting to lighten the motor control requirements of independent sitting when reaching with the other arm (Berger et al., 2019). Similarly, new walkers may use alternative strategies such as crawling or scooting to reach a goal location if the cognitive load is high (Berger, 2010; Berger et al., 2015).

As mentioned above, executive functions show a dramatic improvement in the early childhood period (Anderson & Reidy, 2012; Diamond, 2013; Garon et al., 2008). Core executive function components (i.e., working memory, inhibitory control, set shifting) are thought to support higher‐level executive functions such as planning (Diamond, 2013; McCormack & Atance, 2011; Miyake & Friedman, 2012), suggesting that executive functions might play a role in the choice of motor strategies under different cognitive load. There is also evidence in infancy and childhood that executive functions play a role in the development of hand motor strategies such as fine and gross motor skills (e.g., DeMasi & Berger, 2021; Gottwald et al., 2016; Martzog et al., 2019; Schröer et al., 2022, 2023).

The current research extends these findings into childhood by investigating the use of unimanual and bimanual strategies in a block‐stacking task under varying cognitive load conditions. More specifically, while there is ample evidence that children can use the motor strategy of asymmetrical bimanual coordination by 3 years of age, it is unclear whether the use of this motor strategy is modulated by the cognitive load of the task the child is engaged in. Therefore, this study investigates the influence of cognitive load—in terms of planning complexity—on the use of motor strategies in block‐stacking actions in preschoolers. Experiment 1 describes a mixture of secondary data analysis and new empirical data investigating 3‐year‐old's motor strategies of bimanual coordination or unimanual control in low, medium and high cognitive load stacking tasks. Experiment 2 follows up Experiment 1 using secondary data analyses to investigate how the effect of cognitive load on motor strategy use in 3‐year‐olds changes in 4‐ and 5‐year‐olds, and more specifically whether any such change relates to developing executive functions.

## EXPERIMENT 1

### Method

The data reported in this study is a combination of the recording of videos collected as part of Schröer et al. (2021) investigation assessing hierarchical action sequence planning in preschoolers and new empirical data.

#### Design and participants

The study consists of a mixed within‐ and between‐subject design. Twenty‐five 3‐year‐olds (*M* = 39.24 months, *SD* = 3.36, range = 36 to 46 months, 10 females) participated in the high cognitive load task. These data were obtained from Schröer et al. (2021) Duplo house‐building sample. A further twenty‐five 3‐year‐olds (*M* = 41.52 months, *SD* = 3.87, range = 36 to 46 months, 17 females), drawn from the same participant pool as those from the Schröer et al. (2021) study, participated in the medium and low cognitive load construction tasks. Data collection from the 3‐year‐olds in low and medium cognitive load conditions was carried out in the immediate afterwards of the COVID‐19 pandemic, limiting sample size possibilities. A post‐hoc power analysis using G*Power (including adjusted *α* = .0167) demonstrated that we are able to investigate large effects of Cohen's *d* = .94 at a power of .80 and large effects of Cohen's *d* = 1.37 at a power of .99 in the between‐subject comparison, and medium effects of Cohen's *d* = .69 at a power of .80 and large effects of Cohen's *d* = 1.00 at a power of .99 in the within‐subject comparison.

All participants were typically developing children recruited from the institution's database of interested caregivers. Caregivers gave written informed consent, and children gave verbal assent for participation. Travel expenses were reimbursed, and participants received a certificate and a small gift for participation. A further 10 adult participants, whose data are reported in Supporting Information A, also completed the study. All procedures including secondary data analysis were approved by the local ethics committee.

#### Tasks

All participants completed one or more of the three tasks described below. All three tasks involve identical action—picking up a Duplo block and stacking it on top of another Duplo block—and therefore identical motor demands. The tasks differ in their associated cognitive demands.

##### High cognitive load task

Participants stacked Duplo blocks as part of a planning task. The task involved constructing a Duplo house with a hierarchical goal structure based on an instruction video (Schröer et al., 2021). Children were instructed to pay close attention and build the house the exact same way as in the video; they were asked about the goal and action sequence prior to starting the task. Duplo blocks used for the action sequence were stored in boxes that were mechanically wired to a button. The button could be pressed to open the boxes, enabling children to grasp a Duplo block that could be used to build the house. The house consisted of two walls (6 yellow blocks for first wall, 4 blue blocks for second wall) and a green roof plate. We consider this task to be of (relatively) high cognitive load as it requires participants to maintain a high‐level goal and an action plan appropriate for achieving that goal throughout the task.

##### Medium cognitive load task

Participants were given a two‐block‐high yellow tower and a two‐block‐high blue tower. Participants were then given (in sequence) a yellow window, two yellow blocks to put on top of the yellow tower, and a blue window and two blue blocks to put on top of the blue tower. Lastly, they were given a green plate to put on top of the two towers. Throughout, no specific higher‐level goal was given. Rather, participants were given step‐by‐step construction instructions. We consider this task to be of medium cognitive load because it does not require participants to maintain a high‐level goal and yet the structure produced is identical to that produced in the high cognitive load task.

##### Low cognitive load task

Participants were given a tower of three Duplo blocks and then given one additional Duplo block which they were instructed to place on top of the tower. After completing this, participants were given another block and instructed to place it on top of the growing tower. This process was repeated until the tower was 13 blocks high. We consider this task to be of low cognitive load as participants were provided with instructions after placing each block and no higher level goal was provided.

#### Procedure

**FIGURE 1:**
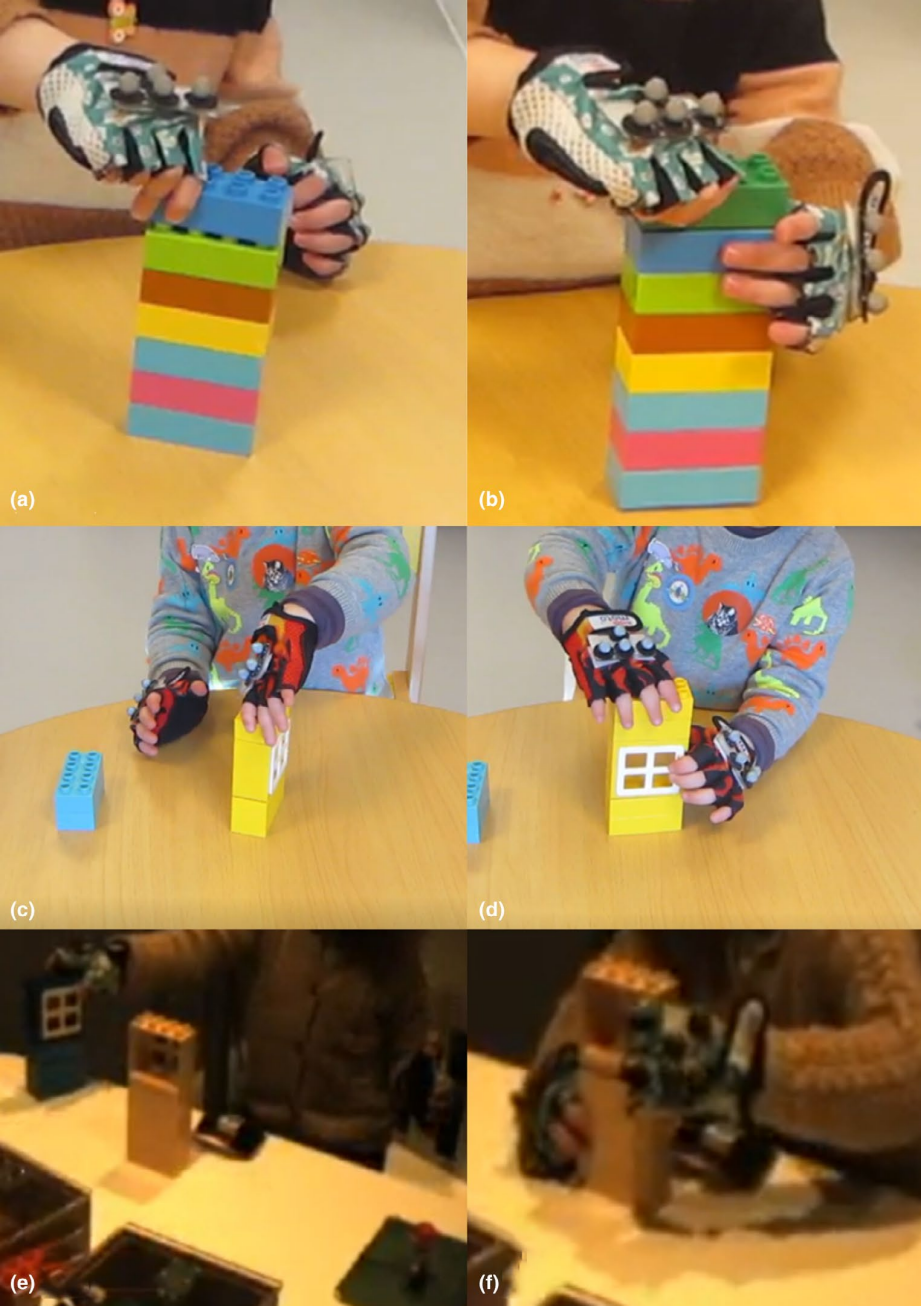
Example stacking in the low (a, b), medium (c, d) and high (e, f) cognitive load task. Left side (a, c, e): Example of unimanual strategy; Right side (b, d, f): Example of bimanual coordination. The video quality in the high cognitive load task (e, f) is poorer as this task was only video recorded using the integrated camera within the 3D motion capture system.

Twenty‐five 3‐year‐olds completed the high cognitive load task as part of a larger motion capture study (Schröer et al., 2021). A further twenty‐five 3‐year‐olds completed the medium and low cognitive load construction tasks. All participants were asked to wear cycling gloves with small plastic plates of reflective marks on both hands throughout the task(s), as shown in Figure 1. For the high‐load task, this was to enable capture of kinematic data (analysed elsewhere). For the medium‐ and low‐load tasks, this was to ensure comparability with the high‐load task. All but two participants wore the gloves in the high‐load condition, while all but four participants wore them in the medium‐ and low‐load conditions.

#### Coding

In each of the tasks, motor strategy for coordination during block stacking was assessed in video recordings of the task. Each block‐stacking action was categorized as *unimanual*, *uni‐to‐bimanual* or *bimanual*. A *unimanual strategy* was defined as stacking a block with one hand, while the other hand was inactive, for example resting by the participant's side or on their lap, even though it would have been useful for completing the action (Figure 1, left side). The unimanual strategy was only counted for blocks that were stacked on another block if, for example, a participant added a block without using their second hand to support the action. *Uni‐to‐bimanual coordination* was coded when the participant's second hand was used after initially failing to use it. *Bimanual coordination* was coded when the participant used their second hand to stabilize the construction (e.g., to push the top‐most block down or place the block correctly—Figure 1, right side). A second coder double‐coded 8 participants of the low and medium cognitive load condition and 8 participants of the high condition. There was very good agreement (Cohen's *κ* = .812). As each participant produced a different number of actions in the high cognitive load task, proportion scores for each of the tasks were used in the further analysis.

### Results

**FIGURE 2:**
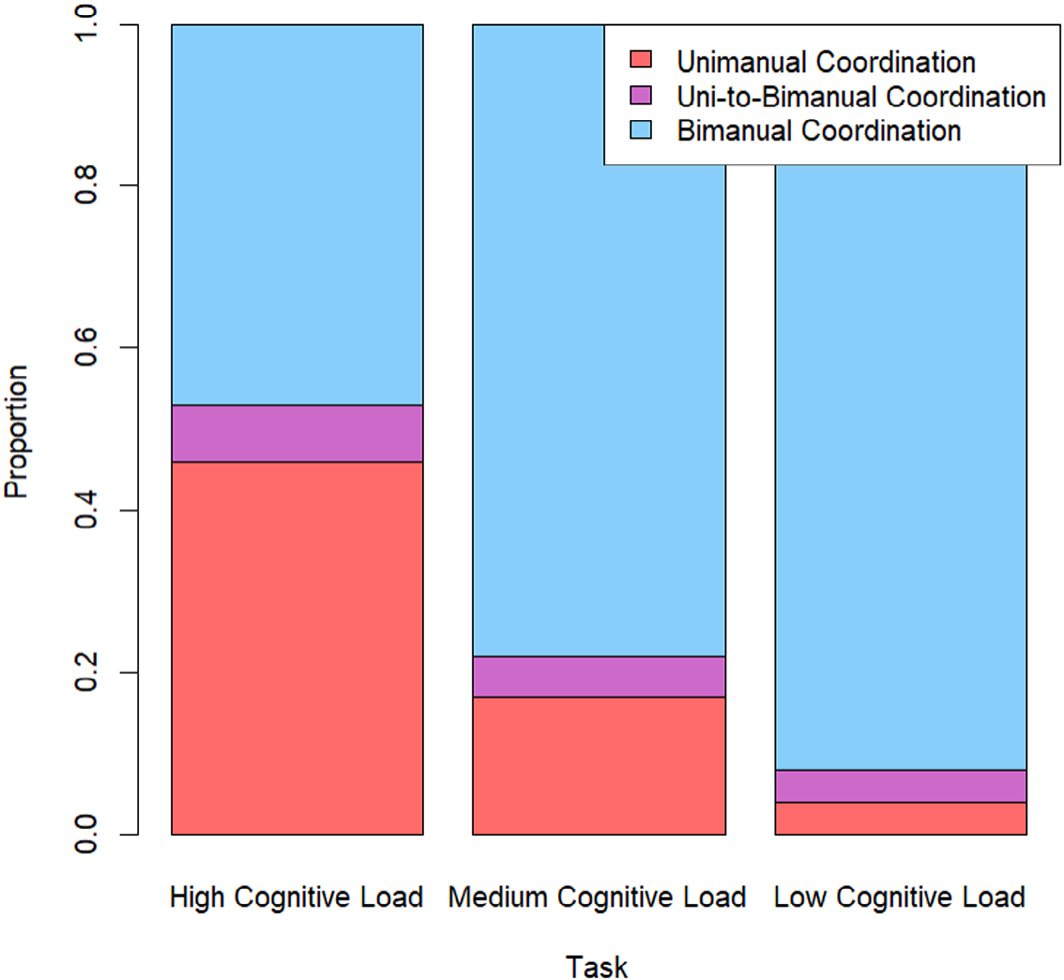
Proportion coordination strategy (unimanual, uni‐to‐bimanual or bimanual) on all tasks (high, medium and low cognitive load) in 3‐year‐olds.

Figure 2 shows the proportion of children who showed bimanual coordination, uni‐to‐bimanual coordination and unimanual strategy use in each of the three cognitive load conditions. As noted above, the current study combined newly collected data (for medium‐ and low‐load conditions) with previously collected data (for the high‐load condition). The comparison between performance in the high cognitive load condition and both the medium and the low cognitive load conditions exploits a between‐subject design and was investigated with independent sample *t*‐tests, whereas the comparison of strategy between medium and low cognitive load condition exploits a within‐subject design and was investigated with a paired‐sample *t*‐test. The critical value of *α* was adjusted for multiple comparisons by Bonferroni correction per outcome measure (*α* = .0167).

Comparing performance in the high cognitive load task with that of the medium cognitive load task (using independent sample *t*‐tests) showed that the proportion unimanual strategy was significantly higher in the high cognitive load task (*M* = 0.46, *SD* = 0.35) than in the medium cognitive load task (*M* = 0.17, *SD* = 0.19) (*t*(48) = 3.65, *p* = .001, Cohen's *d* = 1.03). Correspondingly, the proportion bimanual coordination was significantly lower in the high cognitive load task (*M* = 0.47, *SD* = 0.35) than in the medium cognitive load task (*M* = 0.78, *SD* = 0.22) (*t*(48) = 3.74, *p* = .001, Cohen's *d* = 1.06). The proportion uni‐to‐bimanual coordination did not differ significantly across tasks (*t*(48) = 0.78, *p* = .443).

Comparing performance in the high cognitive load task with that of the low cognitive load task (again using independent sample *t*‐tests) showed that the proportion unimanual strategy was significantly higher in the high cognitive load task (*M* = 0.46, *SD* = 0.35) than in the low cognitive load task (*M* = 0.04, *SD* = 0.14) (*t*(48) = 5.14, *p* < .001, Cohen's *d* = 1.46). Correspondingly, the proportion bimanual coordination was significantly lower in the high cognitive load task (*M* = 0.47, *SD* = 0.35) than in the low cognitive load task (*M* = 0.92, *SD *= 0.20) (*t*(48) = 5.19, *p* < .001, Cohen's *d* = 1.47). The proportion uni‐to‐bimanual coordination did not differ significantly across tasks (*t*(48) = 0.91, *p* = .365).

Finally, comparing performance in the medium cognitive load task with that of the low cognitive load task (using paired‐sample *t*‐tests) showed that the proportion unimanual strategy was significantly higher in the medium cognitive load task (*M* = 0.17, *SD* = 0.19) than in the low cognitive load task (*M* = 0.04, *SD* = 0.14) (*t*(24) = 2.65, *p* = .014, Cohen's *d* = .53). However, the difference in proportion bimanual control in medium‐ and low‐load tasks did not reach significance given the correction for multiple comparisons (*t*(24) = 2.51, *p* = .019, Cohen's *d* = .50). As in previous analyses, there was no significant difference in uni‐to‐bimanual coordination across load conditions (*t*(24) = 0.65, *p* = .524).

### Discussion of experiment 1

While previous studies (e.g., Birtles et al., 2011; Fagard & Jacquet, 1989; Ramsay & Weber, 1986) have demonstrated that preschoolers are able to use both hands in different roles when executing an action (asymmetrical bimanual coordination), the findings in Experiment 1 demonstrate that in a high cognitive load task, preschoolers may use a less efficient unimanual strategy in their stacking actions, even when bimanual coordination would be a more appropriate and more effective course of actions. The use of a unimanual strategy in this high cognitive load task instead of a bimanual coordination strategy can be explained in four possible ways: (i) 3‐year‐olds may struggle to coordinate both of their hands while building with blocks, (ii) the gloves may have impaired 3‐year‐olds' bimanual coordination, (iii) the results may reflect differences between the group of participants used in the Schröer et al. (2021) study and the group recruited specifically for the low and medium cognitive load conditions or (iv) the additional cognitive demands of the action sequence task may have resulted in the use of a different motor strategy to cope with the increased cognitive demands; something that is consistent with the cognitive/action trade‐off hypothesis proposed originally by Boudreau and Bushnell (2000).

As previous studies (e.g., Birtles et al., 2011; Fagard & Jacquet, 1989; Ramsay & Weber, 1986) and performance in the low cognitive load task in Experiment 1 clearly demonstrate, preschoolers can coordinate both of their hands effectively during stacking actions. It is therefore unlikely that 3‐year‐olds struggle in general to coordinate both of their hands while building with blocks (possible explanation i). Furthermore, as the 3‐year‐olds wore the gloves in all three task conditions and used a bimanual coordination strategy in the low and medium cognitive load task, it is also unlikely that the gloves impaired bimanual coordination sufficiently to explain the effect of variable cognitive load on motor strategy (possible explanation ii). Moreover, differences between the groups are unlikely to be the cause of differential strategy use because all participants were drawn from the same database and the age range of the two groups was similar (possible explanation iii). A higher proportion of participants in the high cognitive load condition than the other conditions were male, but there is no reason to believe that sex differences at this age will affect strategy use. Similarly, participants in the high cognitive load condition were on average 2 months younger than those in the other conditions, but this is believed to not account for the magnitude of difference in strategy use between the conditions or that strategy use differed within group in the low‐ and medium‐load conditions, especially considering that the lack of bimanual coordination use in the high‐load condition remains visible over the preschool period (Experiment 2). This makes the cognitive/action trade‐off hypothesis the most likely explanation for performance in the high cognitive load task: 3‐year‐olds used a different and presumably less taxing motor strategy (i.e., a unimanual strategy) because of the high cognitive demands of the planning task.

## EXPERIMENT 2

While Experiment 1 suggests that cognitive load affects motor strategy use in 3‐year‐olds, it does not address the question of how motor strategy use might change over development or of whether individual differences might affect strategy use. Experiment 2 uses data recoded from the Schröer et al. (2021) study of action planning to investigate 3‐, 4‐ and 5‐year‐olds' coordination strategy in a high cognitive load task. Experiment 2 also investigates the mediating role of individual differences in executive function efficacy on the effect of cognitive load on coordination strategy use.

As noted in the introduction, executive functions are cognitive processes that are hypothesized to regulate a person's goal‐directed behaviour (Barkley, 2012; Miyake & Friedman, 2012). Research suggests that the core aspects of executive function (inhibitory control, working memory maintenance/updating and set shifting) improve considerably over the preschool period (Anderson & Reidy, 2012; Diamond, 2013; Garon et al., 2008). Experiment 2 therefore investigated whether improved efficacy of specific executive functions in preschoolers is associated with decreased effects of cognitive load on using the arguably more efficient, but more taxing, strategy ‘bimanual coordination’ when stacking blocks.

### Method

#### Participants

The analyses reported in this study consist of secondary data analysis. A total of forty‐four preschoolers (from Schröer et al., 2021) were included in this study. In addition to the 25 3‐year‐olds considered in Experiment 1 (*M* = 39.24 months, *SD* = 3.36, range = 36 to 46 months, 10 females), there were 24 4‐year‐olds (*M* = 50.71 months, *SD* = 2.79, range = 48 to 58 months, 10 females) and 20 5‐year‐olds (*M* = 63.05 months, *SD* = 2.19, range = 60 to 68 months, 10 females). Two four‐year‐olds provided no data for the task. A post‐hoc power analysis in G*Power showed that we have the ability to detect a medium effect size of *η* ^2^ = .12 at power .80 and a large effect of *η* ^2^ = .26 at power of .99 in ANOVA with three groups to investigate the age effect. Children were typically developing and recruited from the institution's database. All caregivers gave written informed consent, and all children gave verbal assent. Participants received a certificate and a small gift for participation, and travel expenses were reimbursed. All procedures including secondary data analysis were approved by the local ethics committee.

#### Executive function tasks

Computerized tasks were used to assess the core aspects of executive functions (see Schröer et al., 2021). Inhibitory control was assessed with a child‐friendly version of the go/no‐go task (e.g., Kaller et al., 2008). Children had to press the space bar if they saw a bat (26 trials), but not if they saw a cat (9 trials). The inhibition error rate (misses and false alarms, as a proportion of trials) was taken as the score of inhibitory control performance.

Working memory maintenance and updating was measured using an auditory reverse digit span task (e.g., Carlson et al., 2002). Fluffy, a bunny on the screen, said a series of digits and children had to repeat these in reverse order. There were three sets of two, three and four numbers. The proportion correct respective of serial order was taken as updating score, while the proportion correct irrespective of serial order was taken as working memory score (Schröer et al., 2021).

The Schröer et al. (2021) study also used an adapted shifting task to assess set shifting, but the score on this task was found to be insensitive to age, raising questions about the validity of the task in measuring set shifting, it is therefore not considered in the analyses here.

#### Procedure

Children were instructed to build a Duplo house with a hierarchical goal structure (see Experiment 1 *high cognitive load task* and Schröer et al., 2021). Although the action context differed between Experiments 1 and 2 in the different cognitive load conditions, the (motor) task assessed remained the same; namely, block stacking. The task involved both maintaining a high‐level goal of building a house and an action plan appropriate for achieving that goal throughout the task. The block‐stacking strategy (proportion unimanual, uni‐to‐bimanual and bimanual coordination) of all children in each of the three age groups was coded from video data as in Experiment 1.

### Results

**FIGURE 3:**
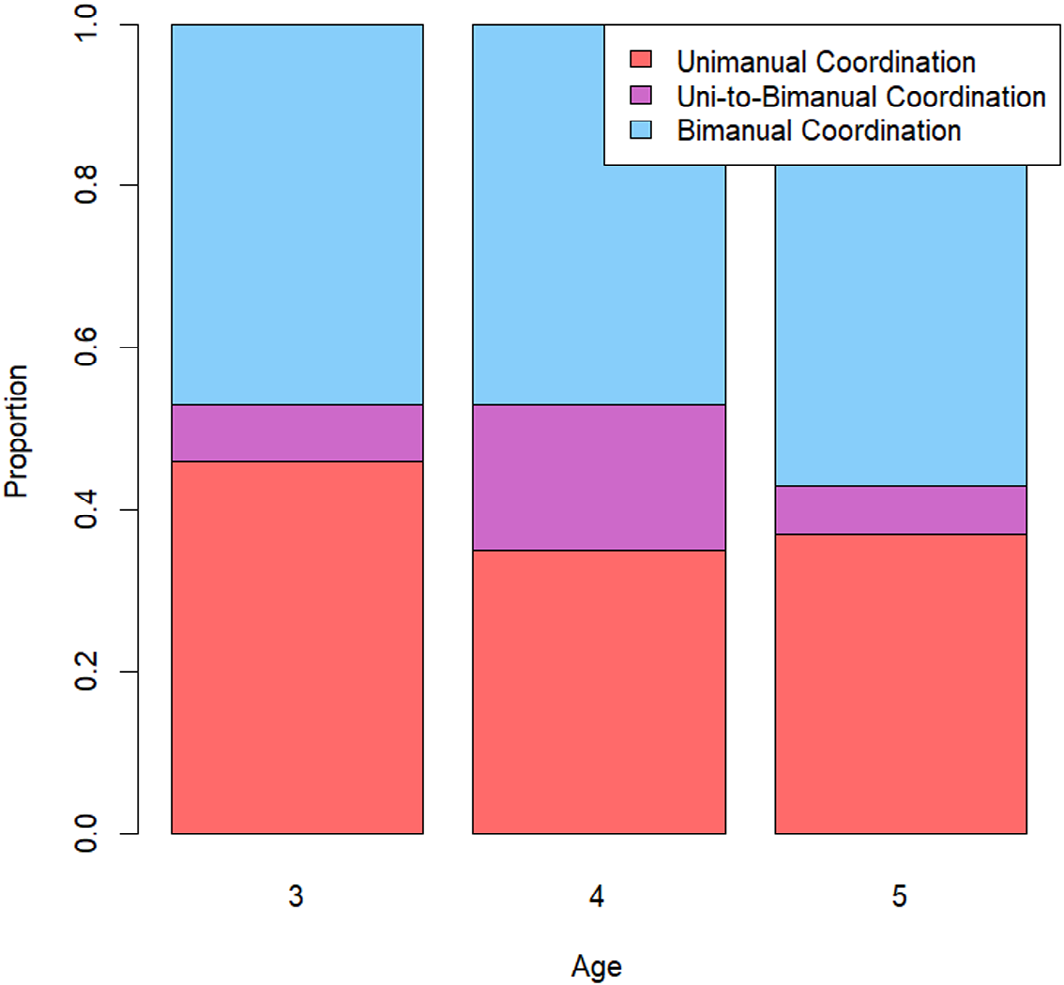
Proportion coordination strategy (unimanual, uni‐to‐bimanual or bimanual) on the planning task with high cognitive load for each age group.

Figure 3 shows the proportion of children who showed bimanual coordination, uni‐to‐bimanual coordination and unimanual strategy use in each of the three age groups. Three one‐way ANOVAs with age group (3 levels: 3‐, 4‐ and 5‐year‐olds) as the between‐subject factor and proportion unimanual strategy, proportion uni‐to‐bimanual coordination and proportion bimanual coordination as dependent variables were performed to investigate the potential age effect on coordination strategy under high cognitive load. There was a significant effect of age on uni‐to‐bimanual coordination (*F*(2,64) = 5.52, *p* = .006, *η* ^2^ = 14). Follow‐up Bonferroni‐corrected tests revealed that 4‐year‐olds showed more uni‐to‐bimanual coordination (*M* = 0.18, *SD *= 0.04) than 3‐year‐olds (*M* = 0.07, *SD* = 0.02) (*p* = .018) and 5‐year‐olds (*M* = 0.06, *SD* = 0.02) (*p* = .014). There was no significant difference for uni‐to‐bimanual coordination between 3‐ and 5‐year‐olds (*p* = 1.00). While the numerical proportion of unimanual strategy use decreased with increasing age, this effect was not statistically significant (*F*(2,64) = 0.79, *p* = .459). Similarly, while the numerical proportion of bimanual coordination increased with increasing age, this effect was also not statistically significant (*F*(2,64) = 0.65, *p* = .526). Thus, it seems that the effect of high cognitive load on strategy use, found with 3‐year‐olds in Experiment 1, persists until at least 5 years of age. Correlation analyses with age in months as a continuous variable showed similar results and are reported in the supplementary materials (Supporting Information B). Although bimanual coordination in 4‐ and 5‐year‐olds was assessed under conditions of high cognitive load, we hypothesize that, like the 3‐year‐olds in Experiment 1, 4‐ and 5‐year‐olds will show increased bimanual coordination in medium and low cognitive load conditions.

**FIGURE 4:**
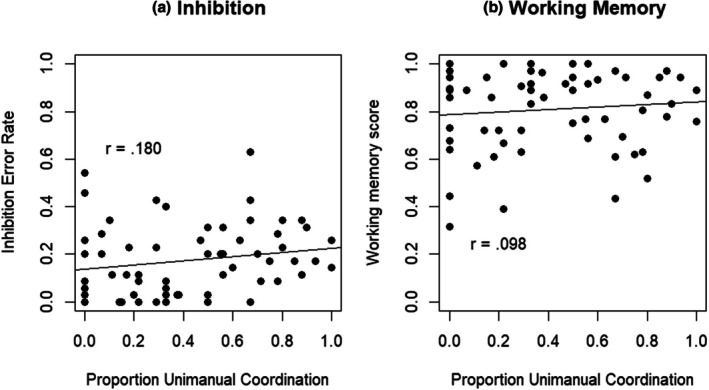
Scatter plots showing the relation between proportion unimanual coordination and (a) inhibitory control score and (b) working memory score. A higher score on inhibition error rate reflects lower inhibitory abilities, while a higher score on working memory reflects better working memory.

To investigate the relationship between increasing executive function efficacy (as executive functions improve with age in the preschool period) in conditions of high cognitive load on coordination strategy, a linear regression with unimanual strategy proportion as the dependent variable and age in months and executive task measures (inhibitory control, working memory and updating scores) as predictors was conducted. This analysis showed that the proportion unimanual strategy can best be predicted by inhibitory control and working memory abilities (*F*(2,59) = 5.29, *p* = .008, Table 1 and Figure 4). Updating score was not a significant predictor and did not explain variance beyond the other factors.

**TABLE 1:** The stepwise linear regression model unimanual strategy use predicted by inhibitory control and working memory.

	*B*	SE	*β*	*t*	*p*
Step 1: Unimanual strategy					.017*
Intercept	0.31	0.06		5.32	<.001*
Inhibitory control	0.66	0.27	0.30	2.46	.017*
*R* ^2^ = .092					
Step 2: Unimanual strategy					.008*
Intercept	−0.13	0.22		0.60	.553
Inhibitory control	0.91	0.29	0.41	3.15	.003*
Working memory	0.50	0.24	0.27	2.05	.045*
*R* ^2^ = .152					

* *p <* .05.

### Discussion of experiment 2

Experiment 2 investigated whether the tendency to use a unimanual block‐stacking strategy in a high cognitive load task changes across the preschool years, comparing 3‐year‐olds' block‐stacking performance to that of 4‐ and 5‐year‐olds in the Duplo house‐building task. The results showed no significant age changes in unimanual strategy use on this task, suggesting that throughout preschool years, children continue to frequently select the (presumably cognitively easier) unimanual strategy when confronted with a high cognitive load task. The lack of effect of age on strategy use might be taken to suggest that differences in strategy use do not occur in this age range. However, contradicting this interpretation, the results also showed that use of the unimanual strategy was predicted by executive function scores; poorer inhibitory control (more errors on the inhibitory control task) was associated with a higher proportion of unimanual strategy use, while, perhaps counterintuitively, a higher working memory score was associated with a higher proportion of unimanual strategy use. This suggests that executive functions affect the choice of motor strategy in high cognitive load tasks in the preschool period.

One way of making sense of the regression results is in terms of the concept of cognitive resource, on the assumption that cognitive tasks draw upon cognitive resource, and hence that performance of a task with high cognitive load will consume a substantial portion of one's available cognitive resource. Given this, the tendency for participants with poorer inhibitory control to make greater use of the unimanual strategy (than those with better inhibitory control) may reflect the fact that poor inhibitory control is associated with greater distractibility. Indeed, the original Schröer et al. (2021) study found that participants with poor inhibitory control were more likely to use distractor blocks in the house‐building task. In order to overcome this tendency to distraction, participants with poor inhibitory control would need to divert more cognitive effort / resource to the primary task, leaving less resource available to support the use of the presumably more taxing bimanual control strategy.

Poor working memory (as reflected in remembering relative few digits, irrespective of order, in the memory task) was also associated with a tendency away from unimanual strategy use (and so towards bimanual coordination). One possibility is that participants with better working memory scores were more engaged with task goals (or better able to direct cognitive resource at those goals) than those with poorer working memory scores, leaving less ‘spare’ cognitive resource in those participants and hence a greater tendency for those participants to revert to the less taxing unimanual strategy. This account is admittedly speculative, as one could also argue that better working memory would leave more resource available for motor control.

A final point from the regression worth noting is that age was found not to be a significant predictor of unimanual strategy use in the house‐building task. While this might seem counterintuitive, it is consistent with the lack of effect of age found in the initial analysis of variance. It is also consistent with previous work demonstrated that age‐related improvement in the performance of a simple goal‐directed action could be accounted for entirely in terms of improvement in executive function and motor competence (Schröer et al., 2022), with age acting as a proxy for development across several variables.

## GENERAL DISCUSSION

Often, when executing actions, we coordinate both of our hands in different or complementary roles. For example, when stacking blocks, we might find it useful to stabilize the tower with one hand, while using the other hand to place the next block on top. Previous studies (e.g., Birtles et al., 2011; Fagard & Jacquet, 1989; Ramsay & Weber, 1986) have shown that this ability develops by the end of toddlerhood. However, in Experiments 1 and 2, we found that preschoolers tended to use a unimanual strategy for block stacking when completing a high cognitive load task—more so than when completing a low cognitive load task.

In Experiment 1, the effect of cognitive load in a motor coordination strategy selection task was investigated. A sample of 3‐year‐olds participated in a low cognitive load task—stacking blocks on a tower—and a medium cognitive load task—stacking blocks on two towers to create a house—and their behaviour was compared with that of a group of 3‐year‐olds when performing a high cognitive load task (planning and executing a hierarchical action sequence) that involved identical stacking actions. The results revealed that 3‐year‐olds showed more unimanual stacking in the higher cognitive load tasks than the lower cognitive load tasks. All 3‐year‐olds showed the ability to coordinate their hands bimanually when stacking blocks in the lowest cognitive load task, despite wearing motion capture gloves. In short, Experiment 1 showed an effect of cognitive load on motor coordination strategy: a cognitive/action trade‐off. Previous studies have shown this cognitive/action trade‐off in infancy (i.e., Berger, 2010; Berger et al., 2018; Berthier et al., 2001; Boudreau & Bushnell, 2000; Hespos & Baillargeon, 2006; Keen et al., 2003) and childhood (Bertrand & Camos, 2015; DeMasi & Berger, 2021; Sebastian & Hernández‐Gil, 2013; Smith et al., 2016).

Experiment 2 investigated whether this effect persisted over preschool years, or whether there was an improvement with age in bimanual coordination strategy use under high cognitive load. Secondary data analysis of 4‐ and 5‐year‐olds' stacking performance showed no significant age effect on bimanual coordination under high cognitive load. However, measures of executive function did associate with motor strategy use, suggesting that differences in executive functions could mediate the effects of cognitive load on the performance of simple actions.

Different studies operationalize cognitive load in different ways. In the postural study of Igarashi et al. (2016), for example, it is operationalized via the contrast between addition/subtraction problems and multiplication/division problems in terms of task difficulty. Cognitive load theory (Sweller, 1988) initially introduced the concept in terms of a task's working memory demands, with the proposal that tasks with high working memory requirements limit learning. Subsequent work distinguished between different forms of load (e.g., perceptual load versus cognitive load) on attentional processing (cf. Lavie, 2005). We have operationalized cognitive load through embedding block stacking within tasks of varying (planning) complexity. The fact that we observed differences in strategy use as a function of our operationalization suggests that the operationalization was successful.

The fact that executive functions mediate simple action planning (i.e., the use of bimanual coordination strategies) is consistent with the idea that core components of executive functions (i.e., working memory, inhibitory control, set shifting) support higher‐level executive functions such as planning (Diamond, 2013; McCormack & Atance, 2011; Miyake & Friedman, 2012). Interestingly, while better inhibitory control skills (i.e., lower error rates on the inhibition task) were associated with reduced use of the less efficient unimanual strategy (an observation consistent with Lavie's, 2005, distinction between cognitive load and perceptual load), better working memory abilities (i.e., recall of more digits, regardless of order, in the reverse span task) were associated with increased use of this strategy. This contrasts with previous studies suggesting that improved working memory leads to better resilience to cognitive load (e.g., Hocking et al., 2020). We can envisage two possible explanations for the results. First, it may be that children with better working memory are able to use bimanual coordination only when it is strictly necessary. We consider this implausible, as (as detailed in Supporting Information A) adults, who generally have better working memory than preschoolers, use bimanual coordination strategies even in the low cognitive load condition. If working memory benefits the ability to selectively use bimanual coordination when strictly necessary, it would be expected that adults also would only use bimanual coordination when strictly necessary. However, the results in our Supporting Information do not support this. Alternatively, it may be that children with better working memory (or more cognitive resource) have the capability to plan and execute their actions according to the goal hierarchy in the high cognitive load task, resulting in reversion to the low‐effort unimanual strategy to compensate for the high cognitive load. In contrast, children with a lower working memory capacity (or less cognitive resource) might instead focus their control processes/resources on the simple motor control task itself instead of the higher‐order planning.

An alternative way to conceptualize the different strategies (unimanual versus bimanual) is in terms of the Norman and Shallice (1986) dual‐systems approach to action selection, in which action is the product of a schema‐based system, *contention scheduling*, that is modulated by the *supervisory system* when deliberate control is required (e.g., when no suitable schema exists). Within this conceptualization, the different strategies are alternative means of achieving the same goal (cf. Cooper & Shallice, 2006). Adults have (presumably) great experience of bimanual stacking and hence have acquired, through this experience, an over‐learned schema within the contention scheduling system. Young children, in contrast, have access only or primarily to a unimanual stacking schema. In order to perform bimanual stacking, they must create and maintain a temporary bimanual schema. This requires inhibitory control (to inhibit the existing unimanual stacking schema) and other executive functions (to build and maintain the temporary schema). The suggestion then is that poor working memory does not prohibit the construction and maintenance of a (temporary) bimanual coordination schema, but good working memory effectively prevents this because it allows supervisory system resources to be directed to the goal of the high cognitive load task. Either way, future research might resolve this by further investigating the relation between cognitive load, task performance and executive functions.

The cognitive/action trade‐off has been used to explain perseveration when task demands are high, errors in means‐end planning tasks and decision‐making, and issues with allocation to resources when attention is loaded (Berger, 2010; Berger et al., 2018; Berthier et al., 2001; Bertrand & Camos, 2015; DeMasi & Berger, 2021; Hespos & Baillargeon, 2006; Keen et al., 2003; Sebastian & Hernández‐Gil, 2013; Smith et al., 2016). These results suggest that if a task is cognitively taxing, more resources are allocated to planning, and less to the relevant motor control. Furthermore, less efficient motor strategies might be used to compensate for the high load while still enabling achievement of the goal (Berger, 2010; Berger et al., 2015, 2019). This is consistent with the findings on postural sway (Blanchard et al., 2005; Igarashi et al., 2016; Olivier et al., 2008; Schmid et al., 2006) and tongue protrusions (Forrester & Rodriguez, 2015) and can be taken as implying that there is a common control system dividing resources between cognitive and motor control processes.

Interestingly, Cognitive load theory in educational science suggests that the use of task‐concordant motor actions or gestures (such as point to a location in space) can reduce cognitive load and improve learning in children (Paas & Sweller, 2011; Sepp et al., 2019). One way of reconciling our findings with those of educational science is to suggest that the use of symbolic actions such as gesturing supports working memory (thereby reducing the load on working memory) and consequently improves learning in high cognitive load conditions (e.g., Goldin‐Meadow et al., 2001; Paas & Sweller, 2011; Sepp et al., 2019). The current study shows that cognitive load has a detrimental effect on basic motor control like bimanual control, and under high cognitive load conditions, preschoolers may be more likely to use inefficient easier strategies such as unimanual control. This suggests a further avenue for future research, namely investigation of the effect of high cognitive load on symbolic actions versus basic motor control.

In summary, the results presented in these studies showed that preschoolers are able to coordinate their hands effectively to stack blocks. However, under a high cognitive load (such as a hierarchical action sequence planning task), a cognitive/action trade‐off occurs resulting in increased use of the easier motor coordination unimanual strategy; this implies a common cognitive control system for cognitive and motor control processes. Preschoolers demonstrated adjustment of their motor strategy use to cognitive load or complexity in the cognitive demands of the superordinate task.

## AUTHOR CONTRIBUTIONS

**Lisanne Schröer:** Conceptualization; writing – original draft; writing – review and editing; formal analysis; project administration; investigation; supervision. **Johanna‐Katharina Maninger:** Conceptualization; writing – review and editing; investigation. **Richard P. Cooper:** Writing – original draft; writing – review and editing; conceptualization; funding acquisition. **Denis Mareschal:** Funding acquisition; writing – original draft; writing – review and editing; conceptualization; supervision.

## CONFLICT OF INTEREST STATEMENT

The authors declare no conflicts of interest.

## Supporting information


Data S1.

## Data Availability

The data that support the findings of this study are available from the corresponding author upon reasonable request.

## References

[bjdp70011-bib-0001] Anderson, P. J. , & Reidy, N. (2012). Assessing executive function in preschoolers. Neuropsychology Review, 22(4), 345–360. 10.1007/s11065-012-9220-3 23109046

[bjdp70011-bib-0002] Babik, I. , & Michel, G. F. (2013). Development of role‐differentiated bimanual manipulation in infancy: Part 1. The emergence of the skill. Developmental Psychobiology, 58(2), 243–256. 10.1002/dev.21382 26644301

[bjdp70011-bib-0003] Barkley, R. A. (2012). Executive functions: What they are, how they work and why. Guilford Press.

[bjdp70011-bib-0004] Berger, S. E. (2010). Demands on finite cognitive capacity cause infants' perseverative errors. Infancy, 5(2), 217–238. 10.1207/s15327078in0502_7 33401790

[bjdp70011-bib-0005] Berger, S. E. , Chin, B. , Basra, S. , & Kim, H. (2015). Step by step: A microgenetic study of the development of strategy choice in infancy. British Journal of Developmental Psychology, 33(1), 106–122. 10.1111/bjdp.12076 25516365

[bjdp70011-bib-0006] Berger, S. E. , Harbourne, R. T. , Arman, F. , & Sonsini, J. (2019). Balancing act(ion): Attentional and postural control strategies predict extent of infants' perseveration in a sitting and reaching task. Cognitive Development, 50, 13–21. 10.1016/j.cogdev.2018.12.001

[bjdp70011-bib-0007] Berger, S. E. , Harbourne, R. T. , & Horger, M. N. (2018). Cognition‐action trade‐offs reflect organization of attention in infancy. Advances in Child Development and Behavior, 54, 45–86. 10.1016/bs.acdb.2017.11.001 29455866

[bjdp70011-bib-0008] Berthier, N. E. , Bertenthal, B. I. , Seaks, J. D. , Sylvia, M. R. , Johnson, R. L. , & Clifton, R. K. (2001). Using object knowledge in visual tracking and reaching. Infancy, 2(2), 257–284. 10.1207/S15327078IN0202_9

[bjdp70011-bib-0009] Bertrand, R. , & Camos, V. (2015). The role of attention in preschoolers' working memory. Cognitive Development, 33, 14–27. 10.1016/j.cogdev.2014.10.002

[bjdp70011-bib-0010] Birtles, D. , Anker, S. , Atkinson, J. , Shellens, R. , Briscoe, A. , Mahoney, M. , & Braddick, O. (2011). Bimanual strategies for object retrieval in infants and young children. Experimental Brain Research, 211, 207–218. 10.1007/s00221-011-2672-5 21499886

[bjdp70011-bib-0011] Blanchard, Y. , Carey, S. , Coffey, J. , Cohen, A. , Harris, T. , Michlik, S. , & Pellecchia, G. (2005). The influence of concurrent cognitive tasks on postural sway in children. Pediatric Physical Therapy, 17(3), 139–193. 10.1097/01.PEP.0000176578.57147.5d 16357673

[bjdp70011-bib-0012] Boudreau, J. P. , & Bushnell, E. W. (2000). Spilling thoughts: Configuring attentional resources in infants' goal‐directed actions. Infant Behavior and Development, 23, 543–566. 10.1016/S0163-6383(01)00059-5

[bjdp70011-bib-0013] Bruner, J. S. (1970). The growth and structure of skills. In K. Connolly (Ed.), Mechanisms of motor skill development (pp. 63–92). Academic Press.

[bjdp70011-bib-0014] Carlson, S. M. , Moses, L. J. , & Breton, C. (2002). How specific is the relation between executive function and theory of mind? Contributions of inhibitory control and working memory. Infant and Child Development, 11(2), 73–92. 10.1002/icd.298

[bjdp70011-bib-0015] Cooper, R. P. , & Shallice, T. (2006). Hierarchical schemas and goals in the control of sequential behavior. Psychological Review, 113(4), 887–916. 17014307 10.1037/0033-295X.113.4.887

[bjdp70011-bib-0016] DeMasi, A. , & Berger, S. E. (2021). Making the process of strategy choice visible: Inhibition and motor demands impact preschoolers' real‐time problem solving. Developmental Science, 24, 313106. 10.1111/desc.13106 33817976

[bjdp70011-bib-0017] Diamond, A. (2013). Executive functions. Annual Review of Psychology, 64(1), 135–168. 10.1146/annurev-psych-113011-143750 PMC4084861 23020641

[bjdp70011-bib-0018] Engström, J. , Markkula, G. , Victor, T. , & Merat, N. (2017). Effects of cognitive load on driving performance: The cognitive control hypothesis. Human Factors, 59(5), 734–764. 10.1177/0018720817690639 28186421

[bjdp70011-bib-0019] Fagard, J. , & Jacquet, A.‐Y. (1989). Onset of bimanual coordation and asymmetry versus asymmetry of movement. Infant Behavior and Development, 12, 229–235. 10.1016/0163-6383(89)90009-X

[bjdp70011-bib-0020] Fagard, J. , & Pezé, A. (1997). Age changes in interlimb coupling and the development of bimanual coordination. Journal of Motor Behavior, 29(3), 199–208. 10.1080/00222899709600835 12453779

[bjdp70011-bib-0021] Forrester, G. S. , & Rodriguez, A. (2015). Slip of the tongue: Implications for evolution and language development. Cognition, 141, 103–111. 10.1016/j.cognition.2015.04.012 25966841

[bjdp70011-bib-0022] Garon, N. , Bryson, S. E. , & Smith, I. M. (2008). Executive function in preschoolers: A review using an integrative framework. Psychological Bulletin, 134(1), 31–60. 10.1037/0033-2909.134.1.31 18193994

[bjdp70011-bib-0023] Goldin‐Meadow, S. , Nusbaum, H. , Kelly, S. D. , & Wagner, S. (2001). Explaining math: Gesturing lightens the load. Psychological Science, 12(6), 516–522. 10.1111/1467-9280.00395 11760141

[bjdp70011-bib-0024] Gottwald, J. M. , Achermann, S. , Marciszko, C. , Lindskog, M. , & Gredebäck, G. (2016). An embodied account of early executive‐function development: Prospective motor control in infancy is related to inhibition and working memory. Psychological Science, 27(12), 1600–1610. 10.1177/0956797616667447 27765900 PMC5154392

[bjdp70011-bib-0025] Hespos, S. J. , & Baillargeon, R. (2006). Décalage in infants' knowledge about occlusion and containment events: Converging evidence from action tasks. Cognition, 99(2), B31–B41. 10.1016/j.cognition.2005.01.010 15939414 PMC1542069

[bjdp70011-bib-0026] Hocking, D. R. , Fritsche, S. , Farhat, H. , Atkinson, A. , Bendak, H. , & Menant, J. (2020). Working memory is a core executive function support dual‐task locomotor performance across childhood and adolescence. Journal of Experimental Child Psychology, 197, 104869. 10.1016/j.jecp.2020.104869 32574754

[bjdp70011-bib-0027] Igarashi, G. , Karashima, C. , & Hoshiyama, M. (2016). Effect of cognitive load on seating posture in children. Occupational Therapy International, 23(1), 48–56. 10.1002/oti.1405 26317316

[bjdp70011-bib-0028] Kaller, C. P. , Rahm, B. , Spreer, J. , Mader, I. , & Unterrainer, J. M. (2008). Thinking around the corner: The development of planning abilities. Brain and Cognition, 67(3), 360–370. 10.1016/j.bandc.2008.02.003 18440114

[bjdp70011-bib-0029] Keen, R. , Carrico, R. L. , Sylvia, M. R. , & Berthier, N. E. (2003). How infants use perceptual information to guide actions. Developmental Science, 6(2), 221–231. 10.1111/1467-7687.00274

[bjdp70011-bib-0030] Kimmerle, M. , Ferrre, C. L. , Kotwica, K. A. , & Michel, G. F. (2010). Development of role‐differentiated manipulation during the infant's first year. Developmental Psychobiology, 52(2), 168–180. 10.1002/dev.20428 20127887

[bjdp70011-bib-0031] Kimmerle, M. , Mick, L. A. , & Michel, G. F. (1995). Bimanual role‐differentiated toy play during infancy. Infant Behavior and Development, 18, 299–307. 10.1016/0163-6383(95)90018-7

[bjdp70011-bib-0032] Kuhtz‐Buschbeck, J. P. , Boczek‐Funcke, A. , Illert, M. , Joehnk, K. , & Stolze, H. (1999). Prehension movements and motor development in children. Experimental Brain Research, 128, 65–68. 10.1007/s002210050818 10473741

[bjdp70011-bib-0033] Lavie, N. (2005). Distracted and confused?: Selective attention under load. Trends in Cognitive Sciences, 9(2), 75–82. 10.1016/j.tics.2004.12.004 15668100

[bjdp70011-bib-0034] Marion, S. D. B. , Killian, S. C. , Naramor, T. L. , & Brown, W. S. (2003). Normal development of bimanual coordination: Visuomotor and interhemispheric contributions. Developmental Neuropsychology, 23(3), 399–421. 10.1207/S15326942DN2303_6 12740193

[bjdp70011-bib-0035] Martzog, P. , Stoeger, H. , & Suggate, S. (2019). Relations between preschool children's fine motor skills and general cognitive abilities. Journal of Cognition and Development, 20(4), 443–465. 10.1080/15248372.2019.1607862

[bjdp70011-bib-0036] Mason, A. H. , Bruyn, J. L. , & Lazarus, J.‐A. C. (2010). Bimanual coordination in children: Manipulation of object size. Experimental Brain Research, 201, 797–807. 10.1007/s00221-009-2100-2 19953229

[bjdp70011-bib-0037] Mason, A. H. , Bruyn, J. L. , & Lazarus, J.‐A. C. (2013). Bimanual coordination in children: Manipulation of object distance. Experimental Brain Research, 231, 153–164. 10.1007/s00221-013-3678-y 23979013

[bjdp70011-bib-0038] McCormack, T. , & Atance, C. M. (2011). Planning in young children: A review and synthesis. Developmental Review, 31, 1–31. 10.1016/j.dr.2011.02.002

[bjdp70011-bib-0039] Miyake, A. , & Friedman, N. P. (2012). The nature and organization of individual differences in executive functions: Four general conclusions. Current Directions in Psychological Science, 21(1), 8–14. 10.1177/0963721411429458 22773897 PMC3388901

[bjdp70011-bib-0040] Norman, D. A. , & Shallice, T. (1986). Attention to action: Willed and automatic control of behavior. In R. Davidson , G. Schwartz , & D. Shapiro (Eds.), Consciousness and self regulation: Advances in research and theory (Vol. 4, pp. 1–18). Plenum Press.

[bjdp70011-bib-0041] Olivier, I. , Palluel, E. , & Nougier, V. (2008). Effects of attentional focus on postural sway in children and adults. Experimental Brain Research, 185, 341–345. 10.1007/s00221-008-1271-6 18214449

[bjdp70011-bib-0042] Paas, F. , & Sweller, J. (2011). An evolutionary upgrade of cognitive load theory: Using human motor system and collaboration to support the learning of complex cognitive task. Educational Psychology Review, 24, 27–45. 10.1007/s10648-011-9179-2

[bjdp70011-bib-0043] Ramsay, D. S. (1985). Infants' block banging at midline: Evidence for Gesell's principle of ‘reciprocal interweaving’ in development. British Journal of Developmental Psychology, 3(4), 335–343. 10.1111/j.2044-835X.1985.tb00985.x

[bjdp70011-bib-0044] Ramsay, D. S. , & Weber, S. L. (1986). Infants' hand preference in a task involving complementary roles for the two hands. Child Development, 57(2), 300–307. 10.2307/1130585

[bjdp70011-bib-0045] Ringenbach, S. D. , & Amazeen, P. G. (2005). How do children control rate, amplitude and coordination stability during bimanual circle drawing? Ecological Psychology, 17(1), 1–18. 10.1207/s15326969eco1701_1

[bjdp70011-bib-0046] Robertson, S. D. (2001). Development of bimanual skill: The search for stable patterns of coordination. Journal of Motor Behavior, 33(2), 114–126. 10.1080/00222890109603144 11404208

[bjdp70011-bib-0047] Schmid, M. , Conforto, S. , Lopez, L. , & D'Alessio, T. (2006). Cognitive load affects postural control in children. Experimental Brain Research, 179, 375–385. 10.1007/s00221-006-0795-x 17136524

[bjdp70011-bib-0048] Schröer, L. , Cooper, R. P. , & Mareschal, D. (2021). Science with Duplo: Multilevel goal management in preschoolers' toy house constructions. Journal of Experimental Child Psychology, 206, 105067. 10.1016/j.jecp.2020.105067 33610884

[bjdp70011-bib-0049] Schröer, L. , Cooper, R. P. , & Mareschal, D. (2022). Left, right, left, right: 24–36‐months‐olds' planning and execution of simple alternating actions. Infancy, 27(6), 1104–1115. 10.1111/infa.12494 35986646

[bjdp70011-bib-0050] Schröer, L. , Cooper, R. P. , & Mareschal, D. (2023). Assessing executive functions in free‐roaming 2‐ to 3‐year‐olds. Frontiers in Psychology, 14, 1210109. 10.3389/fpsyg.2023.1210109 37457086 PMC10338926

[bjdp70011-bib-0051] Sebastian, M. V. , & Hernández‐Gil, L. (2013). Do 5‐year‐old children perform dual‐task coordination better than AD patients? Journal of Attention Disorders, 20(2), 87–95. 10.1177/1087054713510738 24232169

[bjdp70011-bib-0052] Sepp, S. , Howard, S. J. , Tindall‐Ford, S. , Agostinho, S. , & Paas, F. (2019). Cognitive load theory and human movement: Towards an integrated model of working memory. Educational Psychology Review, 31, 293–317. 10.1007/s10648-019-09461-9

[bjdp70011-bib-0053] Smith, A. D. , Gilchrist, I. D. , & Hood, B. M. (2016). Children's search behaviour in large‐scale space: Developmental components of exploration. Perception, 34(10), 1221–1229. 10.1068/p5270 16309116

[bjdp70011-bib-0054] Steese‐Seda, D. , Brown, W. S. , & Caetano, C. (1995). Development of visuomotor coordination in school‐age children: The bimanual coordination test. Developmental Neuropsychology, 11(2), 181–199. 10.1080/87565649509540612

[bjdp70011-bib-0055] Sweller, J. (1988). Cognitive load theory and educational implications. Educational Psychology Review, 2(1), 1–19.

