# Supplementary material for: Bimanual or unimanual stacking strategies under different cognitive loads: Evidence of a cognitive/action trade‐off in the coordination strategy of 3‐ to 5‐year‐olds
Source: Br J Dev Psychol. 2025 Aug 28;44(1):182–98. doi: 10.1111/bjdp.70011 (PMC12884361; doi:10.1111/bjdp.70011)
Supplement: Supplementary file 1 — Data S1. [file BJDP-44-182-s001.docx]

**Supporting Information A – Adult data**

We asked 10 adults (9 females*, M =* 24.95 years old, *SD =* 6.03 years, *range =* 20 to 37 years) to stack blocks according to all the children’s conditions reported in study 1 *(*starting with *low,* then *medium* and lastly *high cognitive load).* Adults wore adult-sized gloves. A repeated measure MANOVA with the three proportion coordination strategy (unimanual, uni-to-bimanual and bimanual coordination) as dependent variables and condition (high, medium and low cognitive load) as within-subject factor showed no significant effects of condition on coordination strategy in adults (*F*(23,7) = 1.56, *p = .*283, Figure S1). Adults used a bimanual coordination strategy to stack blocks, even in the high-cognitive load conditions. Adult used 88% bimanual coordination in the low cognitive load condition, 100% in the medium cognitive load condition and 97.5% in the high cognitive load condition.

**Figure S1**

*Proportion coordination strategy (unimanual, uni-to-bimanual) on all tasks (high, medium and low cognitive load tasks in adults).*


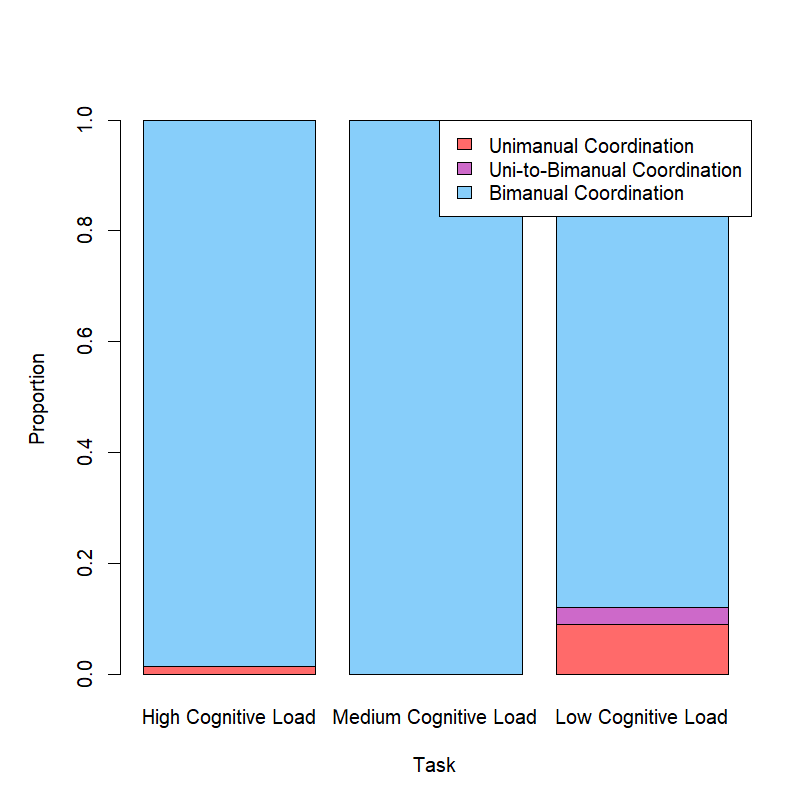


**Supporting Information B – Correlations with age in Experiment 2**

Three Pearson’s correlation analyses were executed to investigate the age effect on proportion coordination strategy (unimanual, uni-to-bimanual or bimanual). There were no significant correlations between age in months (continuous) with proportion unimanual coordination (*r*(64) = -.20, *p = .*113), nor with proportion uni-to-bimanual coordination (*r*(64) = .05, *p = .*714), nor with bimanual coordination (*r*(64) = .17, *p = .*169). The correlation analyses demonstrated that, as described in the result section of Experiment 2 using categorical age bands, there is no significant change with age of the effect on cognitive load on coordination strategy.
